# Muir-Torre Syndrome: case report and molecular characterization: Síndrome de Muir-Torre: relato de caso e caracterização molecular

**DOI:** 10.1590/1516-3180.2014.1321634

**Published:** 2014-02-01

**Authors:** Carolina Alejandra Rios, Ricardo Villalón, Jorge Muñoz, Mónica Acuña, Lucía Cifuentes

**Affiliations:** I PhD. Scientific Researcher, Genetic Epidemiology Laboratory, Department of Human Genetics, School of Medicine, University of Chile, Santiago, Chile; II MD. Attending Physician, Surgical Service, Complejo Asistencial Barros Luco Trudeau, Santiago, Chile; III BSc. Medical technologist, Pathological Anatomy Service, Clínica Dávila, Santiago, Chile; IV MSc. Associate Professor, Department of Human Genetics, Institute of Biomedical Sciences, School of Medicine, University of Chile, Santiago, Chile; V MD, MSc. Full Professor, Department of Human Genetics, Institute of Biomedical Sciences, School of Medicine, University of Chile, Santiago, Chile

**Keywords:** Muir-Torre syndrome, Colorectal neoplasms, hereditary nonpolyposis, Pathology, molecular, Microsatellite instability, Mutation, Síndrome de Muir-Torre, Neoplasias colorretais hereditárias sem polipose, Patologia molecular, Instabilidade de microssatélites, Mutação

## Abstract

**CONTEXT:**

Muir-Torre syndrome is a rare autosomal dominant genodermatosis caused by mutations in the mismatch repair genes. It is characterized by the presence of sebaceous skin tumors and internal malignancies, affecting mainly the colon, rectum and urogenital tract. Awareness of this syndrome among physicians can lead to early diagnosis of these malignancies and a better prognosis.

**CASE REPORT:**

We report the case of a Chilean patient who, over the course of several years, had multiple skin lesions, endometrial cancer and colon cancer. The syndrome was diagnosed using molecular techniques such as microsatellite instability analysis, immunohistochemistry and DNA sequencing, which allowed us to find the causative mutation.

**CONCLUSION:**

Molecular diagnostics is a highly useful tool, since it allows clinicians to confirm the presence of mutations causing Muir-Torre syndrome. It is complementary to the analysis of the clinical data, such as dermatological presentation, presence of visceral malignancies and family history of colorectal tumors, and it provides important knowledge to help physicians and patients choose between treatment options.

## Abstract (pt)

**CONTEXTO:**

A síndrome de Muir-Torre é uma genodermatose autossômica dominante rara causada por mutações nos genes de reparo de incorreções. Caracteriza-se pela presença de tumores sebáceos da pele e doenças malignas internas, afetando principalmente cólon, reto e trato urogenital. A consciência desta síndrome pelos médicos pode levar ao diagnóstico precoce dessas doenças malignas e a um melhor prognóstico.

**RELATO DE CASO:**

Relatamos o caso de uma paciente chilena que, ao longo de vários anos, teve lesões cutâneas múltiplas, câncer de endométrio e câncer de cólon. A síndrome foi diagnosticada com técnicas moleculares, como a análise de instabilidade de microssatélites, imunoistoquímica e sequenciamento de DNA, o que nos permitiu encontrar a mutação causadora.

**CONCLUSÃO:**

Diagnóstico molecular é uma ferramenta muito útil, uma vez que permite que os clínicos confirmem a presença de mutações causadoras de síndrome de Muir-Torre. É complementar para a análise dos dados clínicos, tais como a apresentação dermatológica, a presença de doenças malignas viscerais e história familiar de tumores colorrectais, e fornece conhecimentos importantes para ajudar os médicos e os pacientes a escolher entre opções de tratamento.

## INTRODUCTION

Muir-Torre syndrome (OMIM #158320) is a rare autosomal dominant genetic disease. It is characterized by the presence of sebaceous skin tumors and internal malignancies such as colorectal cancer.^1^ ^,^ ^2^ Muir-Torre syndrome is considered to be a phenotypic variant of Lynch syndrome (OMIM #120435), since these conditions have a common molecular etiology.^3^ ^,^ ^4^

Lynch syndrome accounts for approximately 3% of the incidence of colon cancer worldwide, and can also present with cancer of the urogenital tract, stomach, small bowel, brain and hepatobiliary tract.^5^ ^,^ ^6^ It is caused by germline mutations in genes of the mismatch repair system, mainly *hMSH2* and *hMLH1*. Molecularly, Lynch tumors are characterized by high microsatellite instability and absence of expression of one or more of the proteins that comprise the mismatch repair system.^4^ ^-^ ^6^ These same characteristics are present in Muir-Torre syndrome, but this syndrome differs from Lynch syndrome in that skin lesions are present, such as sebaceomas, sebaceous adenomas and carcinomas, basal cell carcinomas with sebaceous differentiation and seboacanthomas.^7^

Awareness of this syndrome among physicians can help a number of patients and their families, since the proper identification of Muir-Torre syndrome can lead to early diagnosis of visceral malignancies and, hence, better prognosis.

## CASE REPORT

A female Chilean patient consulted the Complejo Asistencial Barros Luco Trudeau in Santiago, Chile, for the first time in 1998 due to a cutaneous lesion. She was diagnosed with a sebaceous cyst. Two years later, the lesion recurred and was excised. The histological analysis showed a well-differentiated sebaceous carcinoma. In 2006, the patient (now aged 49 years) presented stage IIIA endometrial cancer with ovarian metastases. She underwent total hysterectomy and bilateral adnexectomy, together with radiotherapy and brachytherapy, at the same hospital. 

Over the years, the patient had several sebaceous hyperplasias and carcinomas removed. In 2009, Muir-Torre syndrome was suspected by the medical staff at the hospital and she underwent colonoscopy. The patient did not present gastrointestinal symptoms and the carcinoembryonic antigen level was normal (3.0 mg/ml). However, she had a strong family history of colorectal cancer, with one first-degree relative and two second-degree relatives with the disease. 

**Figure 1:**
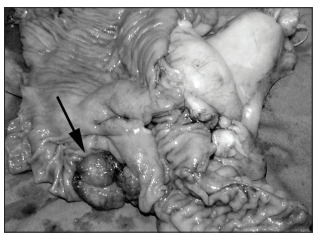
Colon adenocarcinoma.

The colonoscopy detected a lesion in the cecum. The patient underwent right hemicolectomy and received adjuvant chemotherapy (Figure 1). The histology showed a well-differentiated adenocarcinoma with mucinous areas (less than 50%), without lymph node involvement.

**Figure 2:**
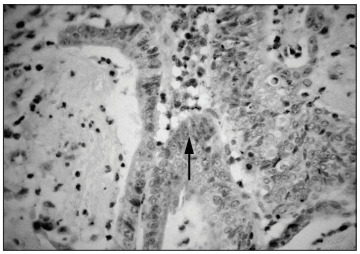
Immunohistochemical staining of colon tissue.

**Figure 3:**
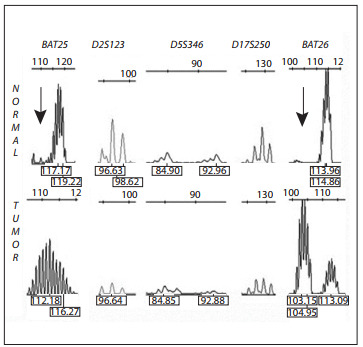
Microsatellite instability analysis.

The genetic testing for Muir-Torre syndrome was performed at the Faculty of Medicine of the Universidad de Chile, Santiago. Paraffin-derived colon tumor tissue was used for all analyses. MLH1 and MSH2 protein expression was studied by means of immunohistochemistry with monoclonal antibodies. Lack of hMSH2 expression was found (Figure 2), while hMLH1 was expressed normally (data not shown). Also, DNA from the patient's colon cancer was tested for microsatellite instability, using the markers BAT25, BAT26, D2S123, D5S346 and D17S250.^8^ The tumor was MSI-high, since it presented over 30% unstable *loci. *In this analysis, both mononucleotide loci (BAT25 and BAT26) were unstable (Figure 3), which is evidence of mismatch repair system malfunctioning. 

Following these results, each exon of the *hMSH2* gene was amplified by PCR and sequenced. The subsequent analysis revealed heterozygote single base pair replacement of cytosine (C) by thymine (T) in exon 13 in position 2131. This nucleotide change causes an alteration in the protein sequence, changing an arginine for a stop codon.

## DISCUSSION

Muir-Torre syndrome is a rare form of Lynch syndrome and is characterized by tumors of the sebaceous glands or keratoacanthoma and a median age of onset of 50 years.^1^ ^,^ ^2^ This syndrome usually arises within families with colorectal cancer histories, but not necessarily histories of skin tumors. Therefore, a key issue in diagnosing Muir-Torre syndrome is the correct identification of any family history of tumors.^2^ ^,^ ^3^

Molecularly, Muir-Torre has a common etiology with Lynch syndrome: germline mutations in the mismatch repair genes. However, different studies have shown that Muir-Torre syndrome is preferentially associated with mutations in the *hMSH2* gene,^1^ ^-^ ^3^ ^,^ ^7^ although mutations in *hMLH1* and *hMSH6* can also be implicated.^9^ ^,^ ^10^ The mutation found in our patient generated a premature stop codon, which produced a truncated MSH2 protein that lacks an important functional domain.^7^ ^,^ ^11^

This syndrome has also been associated with mutations in the MYH gene, which is inherited in an autosomal recessive pattern. These mutations are a cause of attenuated familial polyposis, and the Muir-Torre cases associated with them do not show microsatellite instability.^12^ ^,^ ^13^

Diagnosis of Muir-Torre syndrome is mainly done from the dermatological clinical features and the presence of visceral malignancies or a family history of these.^1^ ^,^ ^2^A systematic search on this topic showed that this syndrome can go unrecognized if clinicians are unaware of these characteristics (Table 1). The role of clinicians in detection and treatment is fundamental. During this search, we found two Brazilian reports of patients with Muir-Torre syndrome.^14^ ^,^ ^15^ Even though the skin lesions presented by these patients were different, they had in common the visceral malignancies and the strong family history, thus highlighting the importance of these clinical characteristics. These two reports did not present any molecular diagnosis, so we were unable to compare them at the molecular level.

**Table 1:**
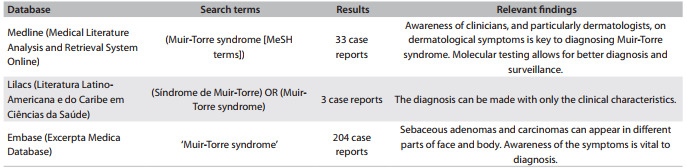
Search strategies regarding the topic of Muir-Torre syndrome. This search was performed on April 8, 2013

The search was narrowed using filters provided by each database: Article type - case reports (PubMed), Tipo de estudio - informe de casos (Lilacs) and Study type - case report (Embase)

Molecular diagnosis has an important role in detecting patients who are carriers of mutations of the mismatch repair system, since such individuals present 80-100% risk of developing cancer.^2^ ^,^ ^5^ Surveillance of these patients and their families is critical, because they can present synchronous or metachronous tumors, especially in the large bowel and the urogenital tract.^16^ Frequent colonoscopies are recommended. This situation is likely to require genetic counseling for the patient and the family, in order to help them understand the nature of the syndrome and the relevance of life-long follow-up.^17^

With regard to treatment, in cases with skin lesions, wide local excision with follow-up is standard for detection of possible metastases. Isotretinoin (retinoid) by itself or combined with interferon has been used to prevent some of the cutaneous neoplasms that have been described.^18^ ^,^ ^19^

Furthermore, use of prophylactic surgery versus endoscopic monitoring has been widely discussed. In patients with colon cancer and proven mutations, total colectomy is recommended, due to the high risk of synchronous and metachronous tumors. However, in patients suspected of having Muir-Torre syndrome, with colon cancer but without a genetic diagnosis, use of prophylactic surgery is controversial.^20^ Women with Muir-Torre syndrome are also at higher risk of endometrial and ovarian cancer. In women of childbearing age, total hysterectomy with salpingo-oophorectomy has been discussed as a prophylactic approach, but there is no consensus on this topic.^21^

## CONCLUSION

Molecular confirmation of Muir-Torre syndrome and Lynch syndrome is an important tool in clinical diagnosis, because it allows discrimination between non-carriers, who do not have an elevated risk of internal or skin malignancies, and mutation carriers, who need strict surveillance in every organ affected by the syndrome.
